# Myeloid cell renin–angiotensin–aldosterone system in hypertension and inflammation

**DOI:** 10.1016/j.cophys.2026.100925

**Published:** 2026-03-12

**Authors:** Claude F Albritton, Sepiso K Masenga, Andrea Marshall, Antentor Hinton, Annet Kirabo

**Affiliations:** 1 Department of Medicine, Division of Clinical Pharmacology, Vanderbilt University Medical Center, Nashville, TN, USA; 2 Department of Molecular Physiology & Biophysics, Vanderbilt University, Nashville, TN, USA; 3 Department of Cardiovascular Science and Metabolic Diseases, Livingstone Center for Prevention and Translational Science, Livingstone, Zambia; 4 Vanderbilt Center for Immunobiology, Vanderbilt Institute for Infection, Immunology and Inflammation, Vanderbilt University Medical Center, Nashville, TN, USA; 5 Vanderbilt Institute for Global Health, Vanderbilt University Medical Center, Nashville, TN, USA

## Abstract

This review explores the intersection of the renin–angiotensin–aldosterone system (RAAS) and myeloid cell biology, with particular focus on how their interaction contributes to hypertension pathogenesis and related inflammatory comorbidities. While the classical RAAS is well-established in systemic blood pressure control and fluid balance, evidence is mounting that immune cells possess an intrinsic RAAS that influences inflammatory processes. We highlight recent findings that myeloid cells not only respond to circulating RAAS factors but also express RAAS components, creating local autocrine/paracrine loops. We also examine emerging links between myeloid RAAS signaling and other conditions such as cardiometabolic disorders, autoimmune diseases, infections, and fibrosis, which suggest broad implications beyond blood pressure regulation.

## Introduction

Hypertension has long been viewed primarily as a hemodynamic disease, but it is now also recognized as a chronic inflammatory condition involving the innate and adaptive immune systems [1]. The renin–angiotensin–aldosterone system (RAAS) is a central hormonal cascade controlling vascular tone and sodium balance. RAAS is a key driver of hypertension and the target of first-line antihypertensive therapies. However, conventional RAAS inhibition often fails to fully control blood pressure and prevent end-organ damage in all patients. This gap has prompted investigation into nonclassical RAAS activity, including within immune cells, that might contribute to ‘residual’ hypertensive risk. Of particular interest are myeloid lineage cells, such as monocytes, macrophages, and dendritic cells (DCs), which infiltrate target organs in hypertension and orchestrate inflammatory injury. Intriguingly, these cells can both respond to RAAS hormones and produce RAAS components themselves [2••,^3^]. A more nuanced understanding of immune cell RAAS signaling in hypertension may uncover new mechanisms of disease and novel treatment strategies.

In this review, we briefly discuss the classical systemic RAAS and its relationship with hypertension before discussing current knowledge of RAAS component expression and function in myeloid cells. We then highlight emerging evidence linking myeloid cell RAAS activity to other disease states beyond primary hypertension. Finally, we offer perspectives on future research directions and therapeutic implications, including the importance of patient-specific factors such as immune status and sex differences.

## Overview of RAAS and its role in hypertension

### Classical RAAS mechanism

**Figure 1:**
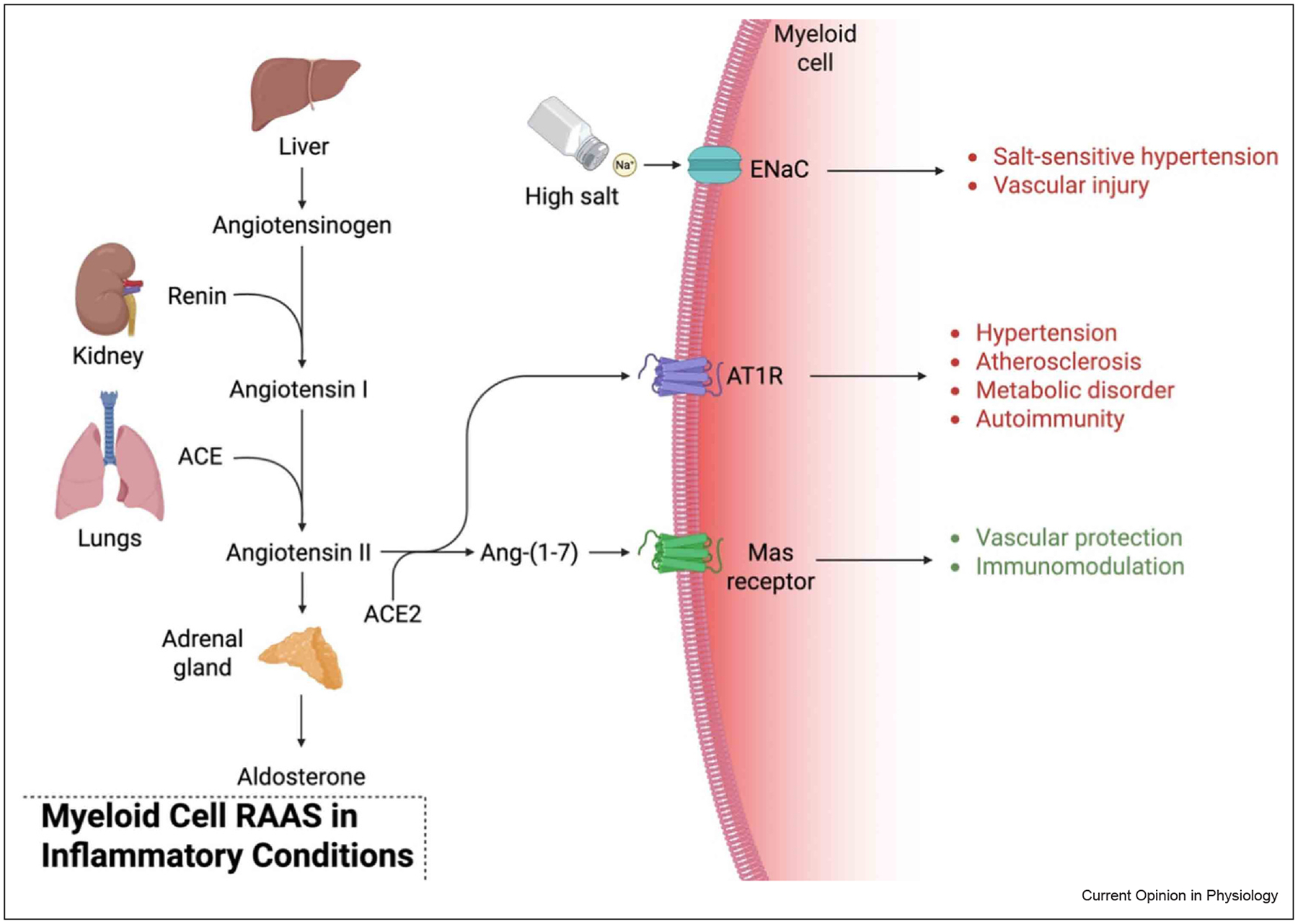
RAAS signaling leading to hypertension. RAAS, renin–angiotensin–aldosterone system; Mas receptor, Mas proto-oncogene G protein–coupled receptor; AT1R, angiotensin II type 1 receptor; ENaC, epithelial sodium channel; Ang-(1–7), angiotensin-(1–7) peptide; ACE, angiotensin-converting enzyme.

The RAAS is a hormone cascade that maintains blood pressure and electrolyte homeostasis. In response to decreased renal perfusion or low distal tubular sodium, the kidneys secrete renin, an enzyme that cleaves circulating angiotensinogen from the liver into angiotensin I. Angiotensin I is then converted by angiotensin-converting enzyme (ACE) primarily in lung endothelium, into the active peptide angiotensin II (Ang II) [4]. Ang II exerts its effects via angiotensin II type 1 receptors (AT1R) and, to a lesser extent, type 2 receptors, on various tissues, causing vasoconstriction, increased sympathetic activity, renal sodium reabsorption, and stimulation of aldosterone release from the adrenal cortex [4]. In myeloid cells, RAAS promotes their activation and contribution to immune-driven hypertension [5,6] (Figure 1). Aldosterone binds mineralocorticoid receptors (MR) in the kidney collecting ducts to upregulate epithelial sodium channels (ENaCs), promoting sodium retention and potassium excretion to restore blood volume [7]. Chronic overactivity of the RAAS leads to hypertension and end-organ damage, which is why RAAS blockade with ACE inhibitors, Ang II receptor blockers, or MR antagonists is a cornerstone of antihypertensive therapy.

### Inflammation in hypertension and RAAS pathology

Beyond hemodynamic effects, Ang II and aldosterone also have potent proinflammatory properties [8]. Hypertension is now regarded as a state of immune activation, where innate and adaptive immune cells infiltrate the vasculature, releasing cytokines and reactive oxygen species (ROS) that drive vascular dysfunction and tissue remodeling [9]. In antigen-presenting cells (APCs), a mechanistic hallmark of this immune activation is the activation of the NLRP3 inflammasome and the formation of isolevuglandins (IsoLGs), highly reactive lipid peroxidation products. These processes lead to the release of proinflammatory cytokines like IL-1β and IL-18 to promote hypertension [10•,^11^]. In fact, NLRP3 inflammasome activation and IsoLG-adduct accumulation in myeloid cells have been directly linked to blood pressure elevation in experimental models [10•,^12^]. As another mediator, the arachidonic acid–derived epoxyeicosatrienoic acids (EETs) appear to have protective effects in salt-sensitive individuals, as higher levels of EETs inhibit myeloid cell sodium channel activation and limit IsoLG production, buffering the hypertensive inflammatory response [13•].

Among immune cells, myeloid lineage cells are particularly important in transducing local tissue signals into systemic inflammation. Macrophages and DCs not only respond to systemic RAAS cues but also express RAAS components themselves, indicating an underappreciated autocrine/paracrine RAAS within immune compartments [2,3]. For example, monocytes and macrophages can express ACE and AT1R on their surface [3]. As major regulators of tissue injury and repair, these myeloid cells significantly influence hypertensive organ damage. Recent single-cell RNA sequencing and proteomic studies have begun to catalog RAAS component expression in myeloid cells from hypertensive animal models and human patients [2]. Interestingly, one study in humans found that acute changes in dietary salt intake (which typically suppress systemic RAAS activity) did not significantly alter RAAS-related gene expression in circulating myeloid cells [2]. This suggests that immune cell RAAS programs may remain active even when the endocrine RAAS is transiently suppressed by high salt, potentially contributing to salt-sensitive hypertension.

Beyond expression profiling, functional studies confirm that myeloid-derived RAAS components are biologically active contributors to blood pressure regulation. For instance, ENaCs on the surface of DCs are critical for sodium-driven inflammatory activation, and blocking these channels reduced inflammasome activity and hypertension in salt-sensitive models [10]. Likewise, Ang II signaling within myeloid cells can directly induce inflammatory gene expression. Using mice with selective overexpression or knockout of ACE2 in myeloid cells, researchers demonstrated that myeloid ACE2 (which converts Ang II to the anti-inflammatory peptide Ang-(1–7)) protects against sepsis-mediated hypotension and vascular dysfunction through the Ang-(1–7)/Mas receptor pathway, dampening NF-κB and STAT1 signaling in macrophages [14•]. As an additional contrast to traditional RAAS ideology, AT1R signaling on DCs can suppress renal DC maturation and T cell activation, preventing sodium retention associated with hypertension [15•]. These findings suggest that RAAS activity within immune cells is not merely a reflection of systemic hormones but rather a functional axis influencing inflammation and blood pressure.

## Activation of myeloid cells in hypertension

Myeloid cell activation in hypertension is multifactorial, involving classical inflammatory stimuli as well as RAAS-driven signals. Ang II and aldosterone can directly activate macrophages and DCs, triggering intracellular pathways that result in oxidative stress, inflammasome activation, cytokine secretion, and enhanced antigen presentation [1]. Ang II–AT1R signaling in macrophages upregulates proinflammatory mediators like TNF-α and IL-1β and ROS production [16]. Ang II can also skew macrophages toward a classically activated M1 phenotype. *In vitro*, Ang II exposure drives human THP-1 macrophages to increase M1 markers like HLA-DR, CD11c, and CD38 while decreasing the M2 marker CD206, an effect largely prevented by AT1R blockade [16]. In parallel, aldosterone signaling in myeloid cells enhances oxidative burst and cytokine release, revealing its key role in driving vascular inflammation. The prohypertensive role of myeloid cells has been further demonstrated through adoptive transfer studies. Such studies noted that the ablation of renal APCs prevented hypertension, cardiac hypertrophy, and perivascular fibrosis while preserving natriuresis in Ang II-infused mice, but adoptive transfer of renal APCs from Ang II-infused mice into wild-type recipients induced a transient increase in blood pressure and reduced natriuresis [17•–19••].

Once activated, nuclear receptor signaling in myeloid cells can further engage pathogenic transcriptional programs. This receptor activation has been shown to cooperate with factors like NF-κB and STAT3 to amplify inflammatory and fibrotic gene expression in cardiac and renal injury models [20,21]. In line with this, MR overactivation in models of heart failure or kidney disease leads to increased inflammatory cell infiltration and upregulation of profibrotic genes, whereas MR antagonists like finerenone, spironolactone, and the novel blocker esaxerenone reduce these deleterious effects [20,21]. In one study, it was found that finerenone and selective myeloid MR ablation protected against chronic dysfunction and fibrosis, which was associated with increased expression of M2 anti-inflammatory markers in macrophages and reduced the expression of proinflammatory markers [22]. Another study concludes that myeloid MR is an important regulator for macrophage polarization, for blood pressure control, and for hypertrophic and fibrotic responses in the mouse heart and aorta [23]. Notably, MR signaling in macrophages contributes to cardiac and renal dysfunction in hypertensive diabetic mice, and myeloid MR deficiency protects against cardiac dysfunction and partially protects against renal dysfunction, highlighting the potential of receptor antagonists in mitigating tissue injury during diabetes [24].

Interestingly, one study claimed that MR signaling in APCs appears to enhance adaptive immunity via DC-specific MR deletion to lower blood pressure and cardiac damage in Ang II–infused mice by blunting IL-6/IL-17A responses [25]. The scientists determined that MR in DCs was found to repress the expression of phospholipase Cβ1/4, which led to hyperactivation of STAT5 and NF-κB that drove pathogenic T helper 17 (Th17) cell differentiation, linking myeloid MR activity to Th17-mediated hypertension [25]. Conversely, a separate study used RNA-sequencing to indicate that *Nr3c2*, the gene for MR, does not exhibit any significant expression in DCs and suggests that the glucocorticoid receptor may be the alternative regulatory nuclear receptor involved [26••]. These stark contrasts present critical quandaries to investigate when considering fundamental differences between renal and immune regulations of hypertension.

Ang II type 1 receptor signaling in myeloid cells likewise contributes to hypertension-related inflammation. Notably, specific deletion of myeloid AT1R can attenuate vascular injury, reduce macrophage recruitment, and improve endothelial function [27••]. Myeloid AT1R activation stabilizes hypoxia-inducible factor 1α (HIF-1α) and triggers Toll-like receptor 4/NF-κB pathways in macrophages [16]. This crosstalk promotes a feed-forward inflammatory state. Consistently, specific deletion of AT1R in myeloid cells markedly attenuated Ang II or deoxycorticosterone/salt-induced vascular injury in mice. AT1R-deficient myeloid cells produced less superoxide, and treated mice showed reduced vascular oxidative stress, fewer infiltrating inflammatory macrophages, and improved endothelial function despite similar blood pressure [27••]. By contrast, a separate mouse study found that AT1R deficiency in bone marrow–derived cells had an augmented hypertensive response compared to the wild-type mouse variants, suggesting a protective role of AT1R on immune cells in hypertension [28]. It is possible that AT1R signaling blockade can inadvertently disrupt its protective role, which could potentially provide an avenue of study for explaining why some hypertensive patients can develop drug resistance to RAAS inhibitors. Notably, aldosterone has been shown to induce NLRP3 inflammasome activation in macrophages, leading to the release of mature IL-1β and sterile inflammation in cardiovascular tissues [10,21]. Taken together, these findings illustrate that RAAS components Ang II and aldosterone activate myeloid cells and downstream HIF-1α, NF-κB, and inflammasome pathways, thereby promoting the chronic inflammation and tissue remodeling characteristic of hypertension.

## Myeloid cell RAAS in various physiological and pathological conditions

### Salt-sensitive hypertension

Salt-sensitive hypertension is a subtype in which blood pressure is abnormally responsive to dietary salt intake. High-salt diet typically suppresses systemic RAAS activity, yet salt-sensitive individuals often still exhibit hypertension accompanied by inflammation. There is growing evidence that RAAS signaling within immune cells sustains inflammatory responses even when endocrine RAAS is low [2]. In mouse models of salt-sensitive hypertension, myeloid cell RAAS appears to be a pivotal driver of tissue injury. For example, in DOCA/salt-treated mice, myeloid AT1R knockout did not significantly lower blood pressure but did reduce cardiac and vascular inflammation, oxidative tissue damage, and fibrosis [27••]. The myeloid-AT1R–deficient mice had improved endothelial function and fewer infiltrating inflammatory macrophages [27••]. This indicates that myeloid AT1R signaling is necessary and sufficient to induce the proinflammatory and profibrotic responses underlying salt-induced end-organ damage, independent of systemic pressure changes. Furthermore, immune cell RAAS activity may explain why some individuals remain salt-sensitive despite low circulating renin/Ang II levels. A study by Ertuglu et al. found that monocytes from salt-sensitive hypertensives maintained expression of RAAS genes and proinflammatory outputs during salt loading [2].

Another insight comes from the role of lipid mediators. As noted earlier, 14,15-EET, an arachidonic acid metabolite, can inhibit ENaC-mediated Na+ uptake and IsoLG formation in APCs during high salt exposure [13•]. Salt-sensitive patients tend to have a blunted EET response, leading to unchecked IsoLG generation and immune activation. Enhancing EET signaling or reducing IsoLG adduct formation has been shown to alleviate salt-driven blood pressure increases in experimental models [3,13]. In summary, within salt-sensitive hypertension, myeloid cell RAAS components such as AT1R and ENaC act as mediators that connect high salt intake to inflammatory organ damage. Targeting these immune RAAS pathways might offer new ways to treat salt-sensitive blood pressure elevation.

### Cardiometabolic disorders

RAAS–immune interactions also play a role in cardiometabolic diseases like atherosclerosis, obesity, and insulin resistance. Ang II is not only a vasopressor but also contributes to metabolic inflammation as high Ang II levels have been linked to coronary artery disease, visceral obesity, and type 2 diabetes in humans [16]. In adipose tissue, Ang II and aldosterone promote macrophage polarization toward an M1 proinflammatory state, which can worsen insulin resistance. Conversely, RAAS inhibition often has anti-inflammatory metabolic benefits such as improved insulin sensitivity and reduced adipose inflammation beyond its blood pressure effect. A striking recent study highlighted the complexity of RAAS signals in atherosclerosis. Cao et al. showed that increasing ACE expression specifically in macrophages reduced atherosclerotic plaque burden in mice [29]. Macrophages with high ACE had enhanced peroxisome proliferator–activated receptor α (PPARα) activity and lipid-catabolizing capabilities, leading to improved cholesterol efflux, greater efferocytosis of dead cells, and a fundamentally altered phenotype that resisted foam cell formation [29]. Notably, this atheroprotective effect of macrophage ACE overexpression was largely independent of Ang II levels, suggesting a novel Ang II–independent role of the ACE protein in macrophage lipid metabolism. This finding is of special interest because it challenges the assumption that more ACE is always proatherogenic. Instead, it implies that modifying RAAS component expression in immune cells could sometimes yield unexpected protective outcomes.

Beyond atherosclerosis, RAAS activation in immune cells has been implicated in obesity-related inflammation. In obese mice, deletion of the angiotensin II type 1 receptor–associated protein (ATRAP) in immune cells effectively increases AT1R signaling and worsens insulin resistance and adipose tissue inflammation, whereas enhancing ATRAP to dampen AT1R signaling had the opposite effect [16]. These observations reinforce that Ang II–AT1R pathways in myeloid cells contribute to systemic metabolic derangements. Additionally, sex-specific RAAS-immune differences may influence cardiometabolic risk (discussed further in a later section). Overall, myeloid cell RAAS activity appears to be an important link between visceral inflammation and cardiometabolic disorders, suggesting that immune-targeted RAAS modulation might complement metabolic disease treatments.

### Autoimmune and inflammatory diseases

Chronic autoimmune disorders like rheumatoid arthritis, systemic lupus erythematosus, and multiple sclerosis are accompanied by persistent inflammation in which RAAS signaling can also be involved. Ang II tends to exacerbate adaptive immune responses, in part by facilitating APC activation and T cell polarization toward proinflammatory phenotypes, such as Th17 cells. Recent studies indicate that RAAS inhibition can ameliorate autoimmune pathology. In an experimental autoimmune encephalomyelitis model of multiple sclerosis, blocking Ang II signaling reduced the frequency of autoreactive Th17 cells while increasing regulatory T cells, thereby mitigating disease severity [30]. Similarly, in a mouse model of autoimmune myocarditis, the ACE inhibitor captopril attenuated myocardial inflammation and injury, even without significantly affecting overall T-cell activation or blood pressure [30]. For example, these immunomodulatory effects of RAAS blockade have been noted in other inflammatory conditions as well, attenuating renal damage in lupus nephritis and reducing colitis severity [30]. It appears that Ang II–AT1R signaling in immune cells can amplify autoimmunity by promoting cytokines like IL-6 and IL-17 and by impairing regulatory immune checkpoints, whereas RAAS blockade tilts the balance back toward immune regulation. Thus, components of the myeloid RAAS axis could be attractive targets not only in hypertension but also in autoimmune diseases driven by similar inflammatory mediators.

## Insights for future studies

The identification of a functional RAAS within myeloid cells has expanded our understanding of hypertension and cardiovascular inflammation. RAAS signaling in myeloid cells dynamically influences local tissue inflammation, vascular tone, oxidative stress, and fibrosis. This new paradigm suggests several important directions for future research.

Firstly, targeting immune cell RAAS pathways is plausible. Current RAAS inhibitors act systemically and do not specifically address RAAS activity within immune cells. The evidence that selective myeloid AT1R deletion can reduce organ damage without major changes in systemic blood pressure [25,27••] is encouraging. It raises the possibility of developing therapies that modulate RAAS components in immune cells. Before such strategies can be realized, specific markers or regulatory mechanisms of myeloid RAAS need to be identified. Key transcription factors that govern RAAS component expression in immune cells, like NF-κB and HIF-1α, could be potential targets. In fact, HIF-1α was shown to mediate Ang II’s proinflammatory effects in macrophages [16], and NF-κB is a known downstream effector of AT1R activation in these cells. Targeting these nodes might blunt the deleterious immune activation while sparing necessary systemic RAAS functions.

Another promising avenue relates to biomarkers and patient stratification. The variability in patient responses to RAAS blockade suggests that, in certain individuals, immune-mediated mechanisms play a larger role. It would be advantageous to identify biomarkers that reflect an ‘immune cell RAAS activation’ signature. For instance, elevated AT1R and ACE levels in circulating monocytes have been proposed as markers of heightened inflammatory response in acute hypertension [3]. Such biomarkers could help predict which hypertensive patients might benefit from adjunctive anti-inflammatory or immune-targeted therapies. They could also be used to monitor the efficacy of interventions aimed at immune RAAS, such as a drop in monocyte AT1R expression after treatment.

Another concept is crosstalk with other innate pathways. Myeloid cells integrate multiple signals from the microenvironment. Future studies should explore how RAAS signaling intersects with toll-like receptor (TLR) and NOD-like receptor pathways in these cells. There is evidence that Ang II can potentiate TLR4 signaling in macrophages via HIF-1α stabilization [16] and that ENaC activation can induce NLRP3 inflammasome priming [10]. Understanding these intersections could reveal synergistic targets. For example, combined inhibition of AT1R and a TLR pathway might have additive benefits in reducing inflammation.

Another prospect is the heterogeneity of myeloid RAAS responses. Not all myeloid cells are the same; monocytes, tissue-resident macrophages, and DC subsets may differ in their RAAS activity and function. Single-cell approaches will be vital to delineate which subsets are most RAAS-active in disease conditions. It will also be important to learn how chronic RAAS activation reprograms myeloid cells and whether these changes are reversible.

Finally, integrating sex as a biological variable is essential in future myeloid RAAS research. Premenopausal women are relatively protected from hypertension compared to men, yet women paradoxically have higher rates of hypertensive cardiovascular complications later in life. Moreover, females, including premenopausal women, tend to have greater salt-sensitivity of blood pressure than males [31,32•]. The recent study by Ishimwe et al. identified the arachidonic acid signaling pathway as a contributor to sex differences in salt-sensitive hypertension [31]. Women with high salt intake showed elevated metabolites in the arachidonic acid/epoxyeicosanoid pathway, suggesting distinct regulatory mechanisms possibly involving estrogen–RAAS interactions and immune modulation between sexes. Myeloid cells from females may respond differently to RAAS stimuli than those of males. For example, estrogen can upregulate ACE2 and anti-inflammatory cytokines to potentially counteract some Ang II effects, whereas postmenopausal loss of estrogen might amplify myeloid cell AT1R/NF-κB signaling. Unraveling these differences could lead to sex-specific therapies for hypertension and heart failure.

In conclusion, the interplay between systemic and immune cell RAAS represents a frontier in cardiovascular research. A deeper understanding of how myeloid cell RAAS contributes to hypertension and related inflammatory diseases could pave the way for innovative treatments that more completely reduce risk. Continued interdisciplinary research that spans immunology, endocrinology, and vascular biology is needed to translate these insights into clinical advances. Targeting the immune component of RAAS may offer hope for patients who remain inadequately managed by current therapies, ultimately improving outcomes in hypertension and its many associated diseases.

## Data Availability

No data were used for the research described in the article.
